# Outcome of Initial Endodontic Treatment Performed, by One Specialist, in 122 Tunisian Patients: A Retrospective Study

**DOI:** 10.1155/2018/3504245

**Published:** 2018-07-30

**Authors:** Latifa Berrezouga, Adel Bouguezzi, Mohamed Semir Belkhir

**Affiliations:** ^1^Department of Restorative Dentistry and Endodontics, Dental Clinic of Monastir, University of Monastir, Tunisia; ^2^Department of Microbiology-Immunology, Faculty of Dental Medicine, Monastir, University of Monastir, Tunisia; ^3^Department of Oral Medicine and Oral Surgery, Dental Clinic of Monastir, University of Monastir, Tunisia

## Abstract

**Objective:**

To assess the 6- to 24-month outcome of endodontic treatments performed, by one specialist, and to identify prognostic factors that may influence initial endodontic treatment outcome (IETO).

**Methods:**

One hundred and forty-six patients out of 163 were included. A number of 201 teeth were endodontically treated, and an overall number of 408 canals were obturated. Of these, 165 teeth received initial endodontic treatment (IET). The criteria of the European Society of Endodontology were used to assess the IETO. The level of significance was set at *p* < 0.05%.

**Results:**

Apical periodontitis (AP) was present in 42.5% of all cases, with a PAI >3 in 28.5%. The success rate (SR) was 91.5%. It was significantly higher in vital teeth (97%) than in devital teeth (87.7%) (*p*=0.04); however, a lower SR was recorded in teeth with AP (*p*=0.02). The lesion healed in 60 teeth (85.7%), decreased in size in 4 teeth (5.7%), and increased in size in 6 teeth (8.5%). A higher SR was obtained when a permanent restoration was present (94%) than absent (68.7%) (*p*=0.005).

**Conclusion:**

Within the limitations of the study, pulp and periapical status and permanent restoration are found to be strong outcome predictors.

## 1. Introduction

Pulp and periapical diseases are most commonly due to caries and dental trauma, but dental material toxicity and iatrogenic procedures are also involved [1]. Boykin et al. [2] reported that dental pain and infection are the most predominant reported reasons for which endodontic treatment is performed, 40 and 30%, respectively. Improvements in molecular biology techniques and in the fields related to endodontics lead to a better understanding of the oral microbiota and management of endodontic infections and apical periodontitis (AP) [3–6]. Despite the extensive literature published regarding success and failure related to root canal treatments (RCTs) or endodontic treatment outcomes (ETOs) [7–13], a great variability exists between study protocols, prognostic factors included and data obtained. Indeed, ETs are performed either by endodontists [14–19], postgraduate students [20–23], students [1, 24, 25], or general dentists [26–28].

Consequently, the overall success rate (OSR) of ETs is affected by these variations. It is comparable between endodontists and postgraduate students. Field et al. [14], Imura et al. [16], and Touboul et al. [23] reported OSRs of 89.2%, 91.45%, and 92%, respectively. Higher SRs were found by Fleming et al. [15] in the group of endodontists using either classic or contemporary RCTs, 98% and 96%, respectively. For students under supervision, the ten-year SR was lower, 85.1% [7]. However, 94% of dentists referring patients with an endodontic problem had no postgraduate qualification in endodontics [26].

Based on strict or loose criteria, SRs were ranged from 31% to 96% versus 60% to 100%, respectively [11].

The assessment of clinical and radiographic ETOs is generally based on European and American guidelines [29, 30]. The Orstavik scoring system index for AP (PAI from 1 to 5) denotes the presence or absence of AP before and after treatment completion [31], and the Wu et al. criteria [32] indicate if the tooth is healed, healing, or diseased. AP is the most reported prognostic factor that significantly affects both initial and endodontic retreatment outcomes. When considering pulp and periapical status, Li et al. [33] reported higher SR for vital teeth (95.38%) and teeth without AP (95.24%) compared to necrosed teeth (81.08%) and teeth with AP (73.24%).

Thus, the primary aim of this retrospective study was to assess the 6 to 24 months outcome of endodontic treatments performed by one specialist, at the Dental Clinic of Monastir, Tunisia, and to identify prognostic factors that may influence initial endodontic treatment outcome (IETO).

## 2. Methodology

### 2.1. Patients Studied

One hundred and forty-six patients with complete medical and dental records were included out of 163 treated patients. Patients aged less than 15 years (*n*=5), or with no follow-up recall (*n*=12) were excluded from the study.

From the 230 treated teeth, 29 were excluded. The remaining 201 teeth with an overall number of 408 canals were divided into 165 teeth with IET and 36 teeth with retreatment. Given the limited number of retreated teeth, only teeth receiving IET were considered for statistical analysis in order to identify predictive factors influencing the treatment outcome. All treatments were performed twice a week for 4 years (from 2012 to 2015), in the Endodontic's Department at the Dental Clinic of Monastir, Tunisia.

For ethical considerations, verbal and written consent were obtained from all patients after being informed about treatment outcomes. Preoperative information included demographic data (age and gender), tooth location, number of root canals, diagnosis pulp, and periapical status (vital or irreversibly inflamed pulpitis, pulp necrosis, and apical periodontitis). Intraoperative information was as follows: number of treatment sessions, type and quality of root canal fillings, complications during treatment such as perforation, breakage of files, and flare-ups. Postoperative information concerned coronal restoration and patients' follow-up period.

### 2.2. Treatment Protocol

Patients were referred from general dentists and specialties other than endodontics, as well. All canals were mechanically prepared, under rubber dam isolation, using the Protaper NiTi rotary file system (Dentsply Maillefer, Ballaigues, Switzerland), according to the crown down technique. Irrigation was performed with 3% NaOCl associated with 17% EDTA in narrow or calcified canals. Full-coverage coronal restorations were either accessed through or cut in half and discarded. Ultrasonic inserts (Endo Success Kit, Aceton, UK) were used to vibrate posts when present and remove pulp stones. The working length was established, using an apex locator (Root ZX®, J. Morita Co., Kyoto, Japan) and conventional radiography. Panoramic radiographs as well as CBCT images performed for some patients either for diagnosis or treatment follow-up were not included in the data analysis. Calcium hydroxide applied with a lentulo-spiral was used as an interappointment dressing for teeth treated in 2 sessions. MTA (Pro-Root MTA, Dentsply Tulsa Dental Co; USA) was placed to seal iatrogenic tooth perforations or create an apical plug for immature necrotic teeth. Root canal fillings were performed using vertical compaction of gutta-percha (System B, SybronEndo, Orange, Ca-lif.) and back-filling with the thermoplasticized injectable gutta-percha technique (ObturaII Spartan, Earth City, Mo.) or carrier-based gutta-percha Thermafil® (Thermafil, Dentsply Tulsa Dental Specialties, Tulsa, Okla.).

### 2.3. Patients' Recall

Patients were either previously scheduled to return for clinical and radiographic control or contacted by telephone. Postoperative information was recorded: the recall period, the presence or absence of signs and symptoms, the presence or absence of apical lesion, and the presence and type of restoration.

### 2.4. Radiographic Assessment

Retroalveolar radiographs were either digital or scanned conventional radiographs. Two independent observers, who have received specific training in endodontics, analyzed all pre- and postoperative radiographs after calibration based on 100 reference radiographs. The Photoshop software (Adobe Photoshop.CS, Version 8.0, USA) was used to visualize the periapical region with ×2 magnification. All studied teeth were scored based on the PAI system [31]. A score ≤2 or ≥3 was attributed for healthy or diseased teeth with AP, respectively.

Root canal filling (RCF) was evaluated according to the European Society of Endodontology criteria [29]. RCF is considered adequate when it is dense and homogeneous with a filling material level within 0–2 mm of the radiographic apex; RCF is inadequate if the root canal is underfilled (>2 mm short of the radiographic apex) or overfilled (extrusion of the filling material beyond the apex), and the RCF is inhomogeneous, not dense with presence of voids. For multirooted teeth, the canal presenting the most inadequate RCF is considered. A final evaluation and agreement were done with a qualified endodontist and an oral radiologist.

### 2.5. Criteria of ETO Evaluation

Clinical and radiographic criteria of the European Society of Endodontology [29] were used to assess the ETO. A favorable ETO is considered in the absence of clinical and radiographic symptoms and signs (Table 1). Teeth with AP were classified as healed, healing, or diseased [32].

### 2.6. Data Analysis

All data were analyzed using SPSS for Windows Version 20.0 (SPSS, Chicago, IL, USA). Results were presented as means ± SD and frequencies. Chi-square or Fisher exact tests were used for comparison of qualitative variables. The level of significance was set at *p* < 0.05.

## 3. Results

A very good agreement was achieved between the two observers (*κ*=0.80).  Table 2 represents the univariate distribution of IET and endodontic retreatment (ERT). The study involved 146 patients, 54.8% females, and 45.2% males with a sex ratio of 1.21 and an overall number of treated teeth of 201. Their age was ranged from 15 to 68 years (mean = 36.8 ± 3.9 years). Of them, 122 received IET (83.56%) and 24 received ERT (17.44%). An overall number of 408 canals were obturated.

### 3.1. Initial Treatment

Most patients (*n*=82) were aged less than or equal to 40 years (67.2%), with a slight predominance of women (55.8%). The overall number of treated teeth was 165. Of them 116 (70.3%) were in the maxilla and 49 in the mandible (29.7%). Anterior teeth (*n*=86) were treated in 52.1% and posterior teeth (*n*=79) in 47.9% of the cases. Teeth were either vital or necrosed, 40.6% or 59.4%, respectively. AP was present in 42.5% of all cases, with a PAI = 3 in 14% and >3 in 28.5%.

### 3.2. Endodontic Retreatment

The number of patients receiving ERT was 24 with a total number of 36 retreated teeth and a similar gender distribution (sex ratio = 1). Patients aged less than or equal to 40 years represented 66.7%. Maxillary and posterior teeth were mostly treated, 63.8% and 72.3%, respectively. All teeth were necrosed, half of them had a PAI ≥3.

### 3.3. Endodontic Treatment Outcome

When considering the number of diseased teeth (*n*=17 out of 201), the overall ETO was 91.54%. This outcome was similar in the IET and retreatment, 91.5% and 91.6%, respectively. The number of diseased teeth was 14 and 3, respectively.

### 3.4. Analysis of Prognostic Factors

Given the representative number of teeth included in the initial treatment, bivariate analysis, shown in Table 3, was conducted to identify prognostic factors influencing treatment outcome.

#### 3.4.1. Preoperative Factors

**Figure 1:**
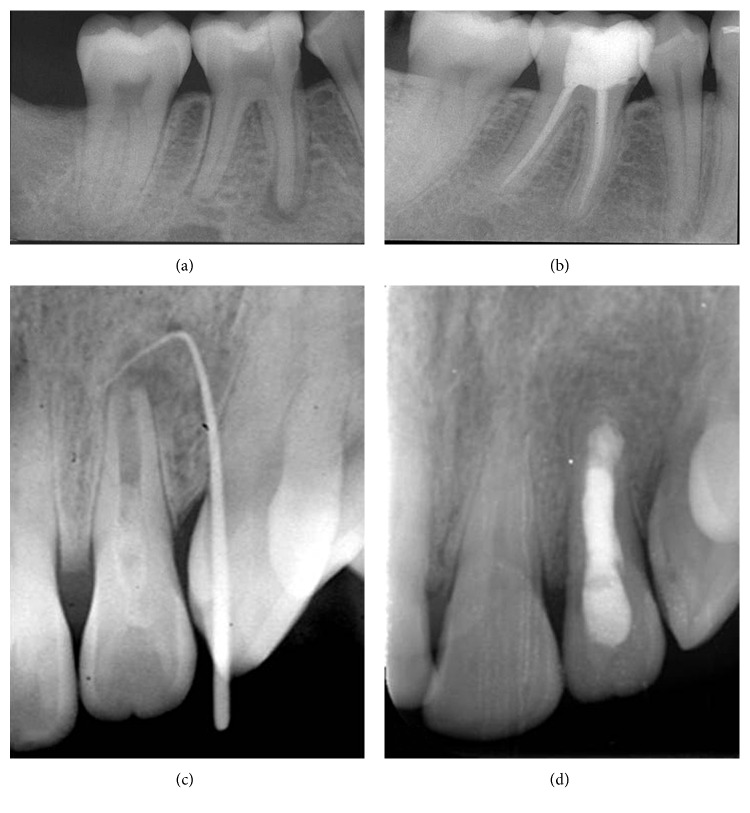
Healed AP. (a) Preoperative view of tooth 46 (PAI = 4), note the resorption of the distal apex; (b) healing at 2-year follow-up (PAI = 0); (c) preoperative view of tooth 22 (PAI = 4), with immature apex; (d) healing at 2-year follow-up, note the apical closure around the MTA plug (PAI = 0).

**Figure 2:**
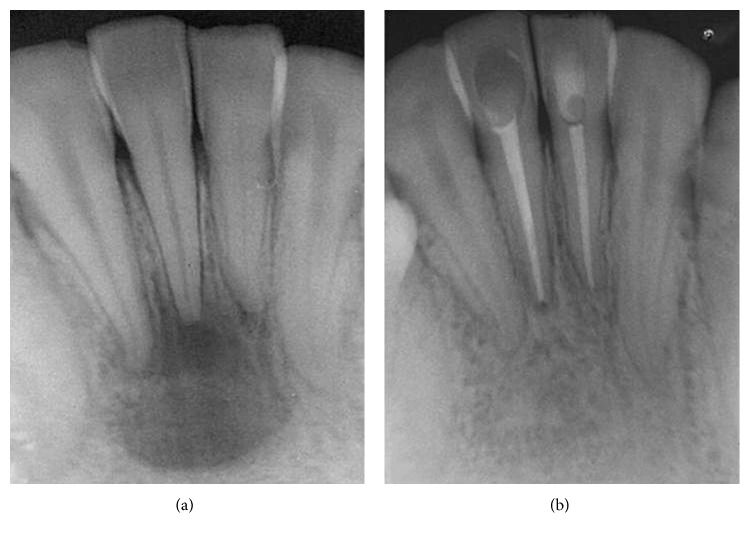
Healing AP on teeth 31, 41, and 42 with a large periapical lesion (PAI = 5). (a) Preoperative view. (b) The lesion is considered healed after 2 years (PAI = 1).

**Figure 3:**
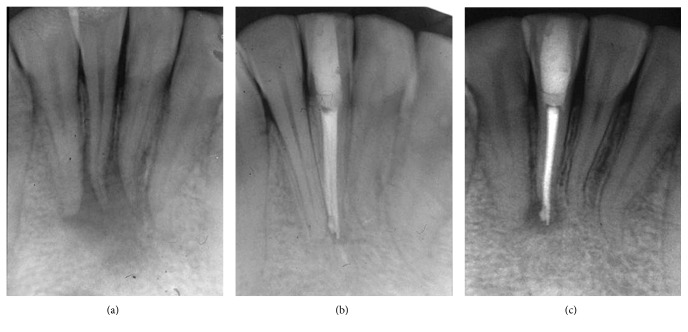
Diseased tooth. (a) Large apical lesion (PAI = 5) in contact of 31, 41, and 42 apices; (b) incomplete healing of the lesion that remained around the apex of tooth 41 in close contact of the overfilling material; (c) note the increase in the size of the lesion.

Although SRs were higher, depending on tooth location, in the maxilla (94%) than in the mandible (85.7%), the difference was not statistically significant. However pulp and periapical status were found to be significant prognostic factors. Among the 165 treated teeth, 67 were vital with a SR of 97% and 98 were nonvital with a lower SR, 87.7% (*p*=0.04, OR = 4.50, 95% CI: 0.95–42.75). Similarly, teeth with no AP displayed significantly higher SRs (95.8%) when compared to teeth with AP (85.7%) (*p*=0.02, OR = 3.76, 95% CI: 1.02–17.18). Of the 95 teeth with no AP, 4 developed AP (PAI ≥ 3); however, in 70 teeth with AP, the lesion healed in 60 teeth (85.7%) (Figure 1), decreased in size in 4 teeth (5.7%) (Figure 2), and increased in size in 6 teeth (8.5%) (Figure 3).

#### 3.4.2. Intraoperative Factors

**Figure 4:**
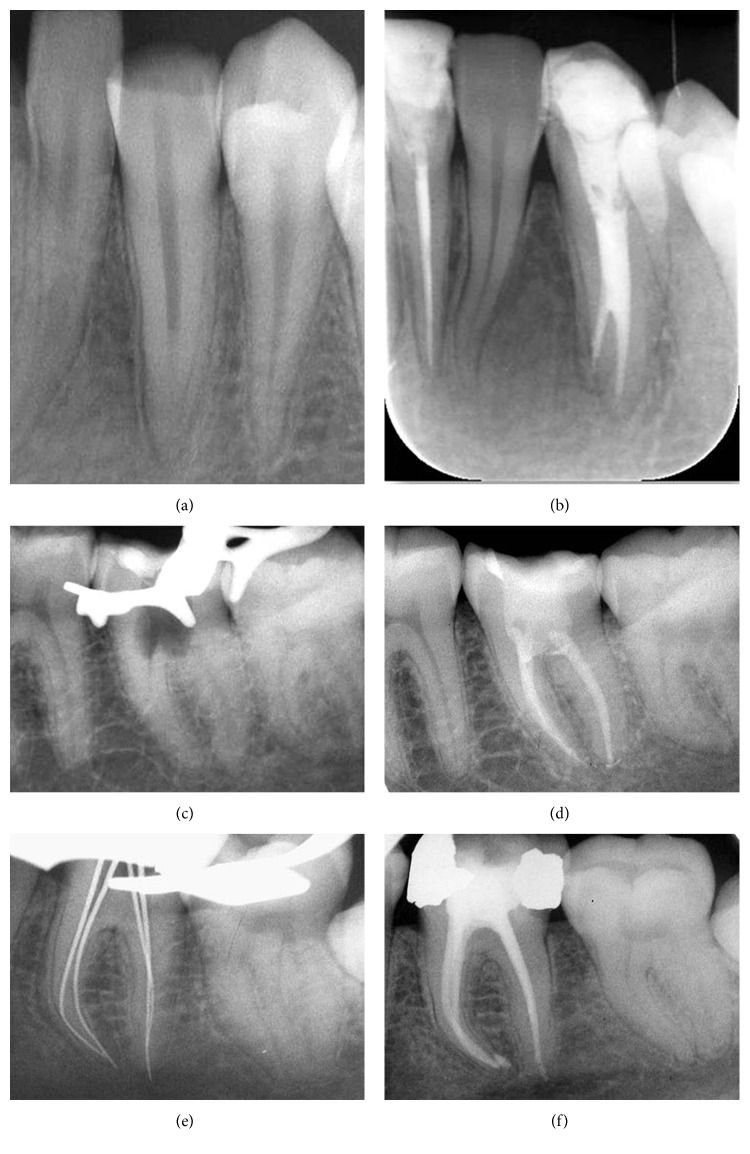
Radiographic views of root canal fillings. (a) Preoperative view of tooth 33; (b) obturation with SystemB/Obtura II; (c) accidental perforation on tooth 37; (d) sealing of the perforation with resin-modified glass ionomer cement and canal filling with SystemB/ObturaII; (e) retroalveolar view of tooth 36; (f) obturation with Thermafil.

No statistical significant difference was observed between teeth treated in one or two sessions. SRs were, respectively, 97.2% and 90%. Complications were seen in 5% of the cases: broken files (1%), accidental perforations (2%), and flare-ups (2%). A higher SR was recorded in teeth where complications were absent than present (96% versus 75%); nonetheless, the difference was not statistically significant. The same finding was observed with the root canal filling level, SRs were higher (92.6%) in teeth adequately obturated (142 out of 165) compared to teeth under or overfilled (82.6%). Figures 4(a)–4(f) show a favorable outcome of teeth obturated using thermoplasticized gutta-percha.

#### 3.4.3. Postoperative Factors

Definitive coronal restorations were performed in 90.3% of the cases, composite resin restorations were mostly used (80%) as compared with amalgam restorations (10.3%). Temporary material filling was performed in 9.7 % of the cases (7.2% for Glass Ionomer Cement and 2.5% for Templin® and Cavit®, resp.). SRs were significantly higher compared to teeth restored with temporary filling material, 94% versus 68.7%, respectively (*p*=0.005, OR = 6.92, 95% CI: 1.55–28.36).

Regarding patients' follow-up, most of them (*n*=76) were seen between 6 and 48 months (62.3%), a total of 114 teeth were reevaluated for the presence of clinical and radiographic symptoms and signs. Of these, 5 teeth remained diseased. However, in the 3- to 4-year follow-up period, the number of patients was lower (*n*=46, 37.7%) with an increased number of diseased teeth (*n*=9).

## 4. Discussion

In the present retrospective study, assessment of clinical and radiographic outcomes of initial endodontic treatments was based on the Endodontic European Society guidelines [29] and Wu et al. criteria [32]. The results revealed that prognostic factors significantly affecting ETO are pulp status, apical periodontitis, and coronal restoration. Gender, tooth location, number of sessions, intraoperative complications, and level of root canal filling are not likely to be associated with ETO.

### 4.1. Pulp and Periapical Status

The present findings are in accordance with those reported by several authors [18, 33–35] stating that ETO is significantly influenced by pulp status (vital versus necrosed pulp) and the presence or absence of a radiolucent lesion. In fact, higher SRs were recorded in vital teeth (97%) and in teeth free of AP (95.8%) than in devital teeth (87.7%). These rates were comparable to those reported by Li et al. [33], 95.3%, 95.2%, and 81%, respectively.

It is well known that infection of the pulp may interfere with ET success, a reason why a more prolonged disinfection is required. However, SRs were significantly lower (85.7%, in the present study) when the infection involves the periapical region, where microorganisms are not efficiently reached and eliminated by conventional irrigants, thereby making AP the main prognostic factor influencing ETO [34]. Healing rates of 82.7% [18], 74%, and 73.2% were recorded by Riccuci et al. [18], Li et al [33], and Friedman et al. [34], respectively. According to Prati et al. [35], a PAI score ≤2 was found to be the predictor of periapical health. That was not the case for some teeth where a new AP appeared (*n*=4) or an initial AP increased in size (*n*=6). This could most probably be due, in the first situation, to underfilled canals (tooth 46) and to root canal reinfection after post-space preparation for prosthetic reasons (3 upper anterior teeth) and to overfilled canals or a persistent AP, in the second situation.

Based on the PAI score system, the prevalence of AP amongst studied patients was relatively high, 42.5%. This could be attributable to the poor oral hygiene of patients seeking dental treatments. By contrast, most studies evaluating AP prevalence were cross-sectional ones based on retrospective radiographic data. Although showing high AP prevalence, these studies cannot determine if a lesion is healing or failing [36–38]. As a matter of fact, it has been demonstrated, over the last years, that CBCT is more sensitive than conventional radiographs in detecting apical lesions and, consequently, avoiding overestimated SRs [39–41]. A significant difference in the overall outcome of both primary and secondary treatments was reported between CBCT (80%) and periapical radiographs (91%) [41].

In the present study, CBCTs used were not included due to the limited number of this 3D radiograph performed for particular conditions (extension of a periapical lesion, complex root canal anatomy, etc.).

Finally, it is worth mentioning that, in 2008, Estrela et al. [42] proposed a new periapical index based on CBCT (CBCTPAI); thus, this radiograph should be used, in long-term longitudinal studies, to evaluate ETO [39].

### 4.2. Coronal Restoration

In vitro studies have demonstrated that bacteria [43] may migrate from the crown to the apex through coronal leakages that expose to tooth reinfection.

There is a general agreement around the relation between coronal restoration quality and ETO. A strong statistically significant difference was found in the present study between definitive and temporary restorations. In fact, higher success rates (94%) were found with teeth adequately restored using composite resin or amalgam. Dawson et al. [44] reported no differences in AP frequencies between teeth adequately restored with amalgam, composite resin, or fabricated full-crowns (i.e., 29.7%, 43.1%, and 26.2%, resp.). An association was found between AP and inadequate root canal filling and marginal bone loss, as well.

Indeed, Tsesis et al. [45] showed that both inadequate root canal filling and coronal restoration may affect the dynamics of AP over a period of 4 years.

### 4.3. Other Reported Prognostic Factors

In the present study, apart from pulp status, AP, and coronal restoration, no other pre-, intra-, or postoperative factors were found to be significant outcome predictors, despite the notable differences seen in SRs. This could be explained by the limited number of teeth treated and the low failure rate recorded (*n*=14). In contrast to what was reported in the literature, quality of root canal filling [46], gender, number of roots, treatment technique of root canal filling, root filling length, and intraoperative complications were significant factors influencing ETO [9, 34, 47]. Moreover, it was clearly shown that higher SRs were reported when ETs were performed by specialists than general dentists or students [19, 48]. An overall SR of 91.5% was found in the present study. As for the number of sessions required to treat teeth with or without AP, it was concluded that there is no clear evidence regarding the benefit of a single-visit versus multiple-visit ET [49].

### 4.4. Patient's Recall

The present findings showed higher SRs for vital and necrosed teeth within 6–12 months recall period, compared with lower rates for teeth with AP. The decrease of SR over time could be explained by a greater number of diseased teeth with AP. Since most teeth with AP heal within one year after endodontic treatment [50], the European Society of Endodontology on quality guidelines recommends at least a year recall after endodontic treatment completion [29]; however, healing of AP can be observed for up to 4 years after the treatment. In the 3- to 4-year follow-up, the recall rate was low (37.7%); nonetheless, this rate was higher (72.85%) given the number of followed teeth with AP (51 out of 70). Completion of treatment, absence of pain, and patient's age, gender, and relocation were factors influencing the recall rate [14]. Some authors like Pirani et al. [46] reported the long-term tooth survival and healing rates, while others recorded functional teeth for a longer period of time with [51] or without AP and adequate restorations [52].

It is in this context that further prospective studies, involving a large number of patients, should be conducted in order to investigate the influence of pre-, intra- and postoperative prognostic factors on both outcome measures: tooth survival and AP healing.

## 5. Conclusion

Within the limits related to the present study, the authors conclude the following:

- The overall success rate is comparable to that reported by endodontists and postgraduate students. A higher SR was obtained in vital pulps compared to teeth with AP. Consequently, pulp and periapical status are strong predictor factors of IETO.
- For teeth free of AP, at least, a 6-month recall is required to clinically and radiographically rule out signs and symptoms of any periapical disease.
- Longer time is needed for teeth with AP, when considering the PAI score index and the low dynamic of the healing process.
- Despite the limited number of teeth included, the high prevalence of AP observed may reflect the low level of oral hygiene of patients seeking dental treatments in the endodontics' department.

## Figures and Tables

**Table 1:** Assessment of root canal treatment outcome according to the European Society of Endodontology [29].

Outcome	Clinical findings	Radiographic findings		Recall period
Favorable	(i) Absence of pain, swelling, sinus tract, loss of function, and other symptoms	*Initial*	*Recall*	At least one year
		(i) Normal periodontal space around the root	(i) Periodontal space unchanged	
		(ii) Radiolucent area	(ii) Healing of the lesion with normal periodontal space around the root	

Uncertain		(i) Radiolucent area	(i) No changes in the size of the initial lesion	At least after 4 years

Unfavorable	(i) Presence of pain, swelling, sinus tract, loss of function, and other symptoms	(i) Periodontal space remained normal after endodontic treatment	(i) Radiolucent area	Further treatments are required
	(ii) Presence of signs of root resorption	(ii) Radiolucent area	(i) ^a^Absence of healing: radiolucent area remained the same, increased, or diminished in size during the 4-year assessment period	

^a^For an extensive radiological lesion, the tooth should be further assessed, because the lesion may heal but form a scar tissue.

**Table 2:** Univariate distribution of endodontic prognostic factors.

Prognostic factors	Initial treatment	Retreatment
	N^a^/%^b^	N^c^/%^d^
Preoperative		
** **Age		
** **≤40 years	82/67.2	16/66.7
** **>40 years	40/32.8	8/33.3
** **Gender		
** **Female	68/55.8	12/50
** **Male	54/44.3	12/50
** **Tooth location		
** **Maxilla	116/70.3	23/63.8
** **Mandible	49/29.7	13/36.2
** **Type of tooth		
** **Anterior	86/52.1	10/27.7
** **Posterior	79/47.9	26/72.3
** **Pulp status		
** **Vital	67/40.6	0/0
** **Nonvital	98/59.4	36/100
** **Periapical status		
** **PAI = 1	85/51.5	18/50
** **PAI = 2	10/6.0	0/0
** **PAI = 3	23/14	10/27.8
** **PAI > 3	47/28.5	8/22.2
Intraoperative		
Treatment session		
** **1	36/21.8	7/19.5
** **2	129/78.2	20/55.5
** **3	0	9/25
Intracanal dressing		
** **Yes	129/78.2	29/80.5
** **No	36/21.8	7/19.5
Root canal filling level		
** **Adequate	142/86	29/80.5
** **Overfilled	15/9.0	3/8.4
** **Underfilled	8/5	4/11.1
Root canal filling density		
** **Yes	158/95.8	35/97.2
** **Voids	7/4.2	1/2.8
Complications		
** **No	157/95	32/89
** **Yes	8/5.0	4/11
Postoperative restoration		
** **Permanent	149/90.3	27/75
** **Temporary	16/9.7	9/25

^a^In the initial treatment, the number of individuals treated is 122 with an overall number of teeth equal to 165; ^b^corresponding rates. ^c^The number of patients receiving endodontic retreatment is 24 with a total number of teeth equal to 36; ^d^corresponding rates.

**Table 3:** Bivariate distribution of initial endodontic prognostic factors.

Prognostic factors	N/%^a^	Success^b^ (N/%)	*p* value^c^	OR^d^	CI_95%_^e^
Preoperative					
** **Gender			1	0.88	0.19–3.27
** **Female	114/55.8	104/91.2			
** **Male	51/44.3	47/92.2			
** **Tooth location			0.12	2.57	0.72–9.19
** **Maxilla	116	109/94			
** **Mandible	49	42/85.7			
** **Vital pulp			**0.04**	4.50	0.95–42.75
** **Yes	67	65/97			
** **No	98	86/87.7			
** **Apical periodontitis			**0.02**	3.76	1.02–17.18
** **No	95	91/95.8			
** **Yes	70	60/85.7			
Intraoperative					
** **Treatment session			0.19	4.01	0.56–176.02
** **1	36	35/97.2			
** **2	129	116/90			
** **Complication			0.13	0.25	0.039–2.81
** **No	151	145/96			
** **Yes	8	6/75			
** **Adequate root canal filling level			0.11	2.75	0.57–10.83
** **Yes	142	132/92.6			
** **No	23	19/82.6			
Postoperative					
** **Restoration			**0.005**	6.92	1.55–28.36
** **Permanent	149	140/94			
** **Temporary	16	11/68.7			

^a^Total number of teeth and correspondent percentages; ^b^number of successful cases and success rates; ^c^Fisher's exact tests are used; ^d^odds ratio; ^e^95% confidence interval. Statistically significant values are shown in bold.

## Data Availability

The data used to support the findings of this study are available from the corresponding author upon request.
